# Application of Mixed Reality Using Optical See-Through Head-Mounted Displays in Transforaminal Percutaneous Endoscopic Lumbar Discectomy

**DOI:** 10.1155/2021/9717184

**Published:** 2021-02-16

**Authors:** Xiaoyang Liu, Jianmin Sun, Meimei Zheng, Xingang Cui

**Affiliations:** ^1^Department of Spine, Shandong Provincial Hospital Affiliated to Shandong First Medical University, Shandong Provincial Hospital Affiliated to Shandong University, Jinan, China; ^2^Department of Neurology, The First Affiliated Hospital of Shandong First Medical University, Jinan, China

## Abstract

**Purpose:**

Mixed reality (MixR) technology merges the real and virtual worlds to produce new environments and visualizations; it is being tested for numerous minimally invasive surgical procedures. This study is aimed at evaluating the use of MixR technology using optical see-through head-mounted displays (OST-HMDs) during transforaminal percutaneous endoscopic discectomy (TPED).

**Methods:**

Forty-four patients treated with MixR-assisted TPED through OST-HMDs were compared with matched patients treated with conventional TPED (*n* = 43). In the MixR-assisted TPED group, MixR technology was used to navigate the four procedures of marking, needle insertion, foraminoplasty, and positioning of the working sheath. The clinical outcomes were evaluated based on the numerical rating scale (NRS) scores and Oswestry Disability Index (ODI) on preoperative and postoperative day 1 and at the last follow-up examination. The procedural times, radiation exposure, and eye fatigue were also recorded. All patients were followed up for at least 6 months.

**Results:**

The NRS scores and ODI were significantly improved in both groups at the last follow-up visit compared with the preoperative values (*P* < 0.05); these values were not statistically different between the groups. The operation time and radiation exposure during marking, needle insertion, and total procedure significantly decreased in the MixR-assisted TPED group compared to those in the conventional TPED group (*P* < 0.05). Unfortunately, the incidence of eye fatigue increased owing to the use of OST-HMDs in the MixR-assisted TPED group.

**Conclusion:**

This study shows the utility of MixR technology for image guidance in conventional TPED. Radiation exposure is decreased, and this technology serves as a valuable tool during the TPED procedure; however, the assistance of conventional fluoroscopy is still required.

## 1. Introduction

Transforaminal percutaneous endoscopic discectomy (TPED) is a typical minimally invasive discectomy procedure. Precise puncture and cannulation are significant steps in TPED, and the achievement of precise puncture and cannulation depends on the surgeon's experience and fluoroscopic guidance [1]. Thus, the surgical process of TPED is reported to have a steep learning curve and involves radiation exposure [2]. Increased radiation exposure may be associated with potential radiation-induced adverse events [3]. Therefore, it is important to reduce the radiation dose of practitioners to minimize the risk of potential radiation-induced complications.

Virtual reality has proven to be feasible in TPED. It enables precise surgical planning and improves intraoperative procedures; therefore, it has the potential for application in clinical practice [4]. Mixed reality (MixR) technology is the merging of real and virtual worlds to create new environments and visualizations where the physical and digital objects coexist and interact in real time [5, 6]. Optical see-through head-mounted displays (OST-HMDs) with high resolution and high contrast capabilities offer real-time MixR visualization of radiographic images that can be projected over the interventional site without hampering direct control of procedural manipulations [7]. MixR devices have been tested in image-guided minimally invasive surgical procedures [6, 7]. It is speculated that MixR technology may improve the surgical experience, shorten the operating time, and decrease the adverse effects of TPED. Unfortunately, MixR through OST-HMDs has not been introduced in TPED. Herein, we attempt to introduce and determine the utility of MixR navigation during the conventional TPED procedure.

## 2. Material and Methods

### 2.1. Patients

This comparative study was approved by the ethics committee of a university hospital and was conducted in accordance with the guidelines of the Declaration of Helsinki. Written informed consent was obtained from each participant. From June 2018 to July 2019, 44 patients with lumbar disc herniation, who had failed to respond to conservative treatment for more than 6 weeks, were treated with MixR-assisted TPED. Another 43 patients were selected from a clinical database between February 2018 and June 2019, according to their demographic characteristics and disease-related features to ensure comparability between the two groups; these patients were treated with conventional TPED. The inclusion criteria were as follows: (1) radicular leg pain due to lumbar disc herniation confirmed through magnetic resonance imaging and (2) TPED at a single level. The exclusion criteria were as follows: (1) segmental instability, (2) lumbar spinal stenosis, (3) calcified disc herniation, (4) recurrent lumbar disc herniation, (5) painless weakness, and (6) TPED at multiple levels. All patients were followed up for at least 6 months, with an average of 12 months.

### 2.2. 3D Virtual Model Reconstruction and Working Plan

**Figure 1:**
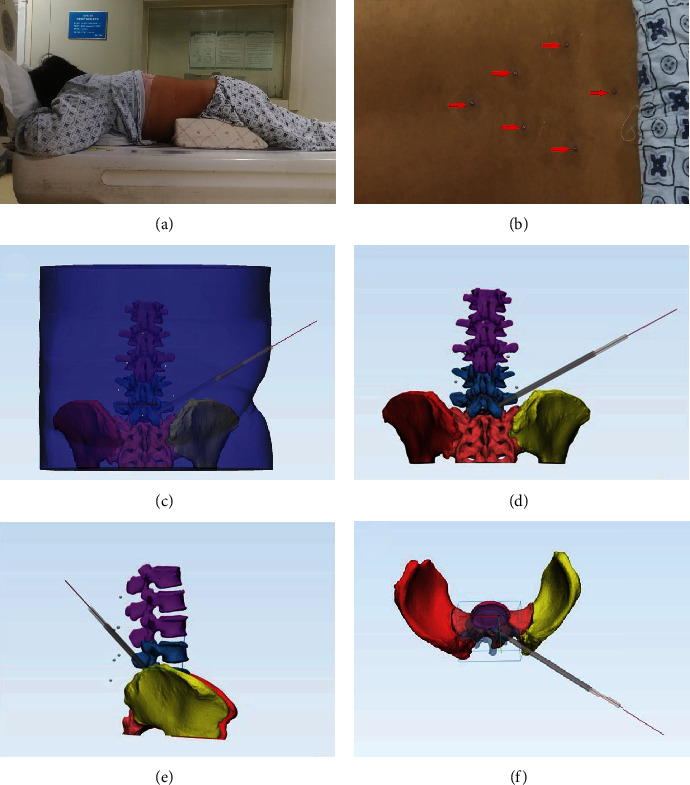
Computed tomography (CT) examination and reconstruction of the three-dimensional (3D) model. (a) Flexion position during lumbar CT examination in which the patient was positioned in the prone position on a hard sponge cushion. (b) External landmarks (red arrows) attached to the skin around the surgical segment during the CT examination; these landmarks were used for preliminary matching. (c–f) 3D model reconstructed from the CT data.

The patient with the diagnosis of lumbar disc herniation confirmed using magnetic resonance imaging was positioned in the prone position on a hard sponge cushion for the lumbar computed tomography (CT) examination preoperatively (Figure 1(a)). While referring to the anteroposterior radiograph, six steel balls with a diameter of 2.5 mm were attached to the skin, close to the surface projections of the S1 and L2 spinous processes, both posterior superior iliac spines, and both transverse tips of the fourth lumbar vertebra (Figure 1(b)). The CT images were acquired using a 64-row CT scanner (Siemens Healthineers, Erlangen, Germany) set at 120 kVp or 140 kVp with an adaptive tube load of 200–300 mAs, depending on the patients' weight and size. After the CT examination, all markers were labeled on the skin and then removed. The original DICOM data were input into Mimics software version 20.0 (Interactive Medical Image Control System, Materialise, Leuven, Belgium) and reconstructed into the 3D virtual model. The soft tissues, vertebrae, discs, nerves, and markers were extracted and colored using the threshold, mask, region growth, and other embedded tools. The puncture target was determined from the preoperative radiographic evaluations. If foraminoplasty was required, the superior articular process of the inferior lumbar vertebra was considered the puncture target. Otherwise, the ruptured disc served as the puncture target. The models of the needle and working sheath were created and placed on the preoperative model (Figures 1(c)–1(f)).

### 2.3. Building a MixR Environment

The Microsoft HoloLens using a Windows Holographic platform under the native Windows 10 operating system was connected to a local area wireless network [8]. The integrated 3D virtual model was loaded into the Scene Editing System(Midivi, Changzhou, China, https://www.midivi.cn). All elements were defined by different colors and transparencies, aimed at optimal visualization. The final 3D model was then exported and loaded into the MixR system(Midivi, Changzhou, China, https://www.midivi.cn) on a computer for processing and uploading into the local network. The OST-HMD (HoloLens 2, Microsoft Corporation, State of Washington, USA) loaded the data and displayed the 3D virtual model. The operators could simultaneously visualize the 3D model, working plan, and procedural site in a MixR environment.

### 2.4. MixR-Assisted TPED Technique

**Figure 2:**
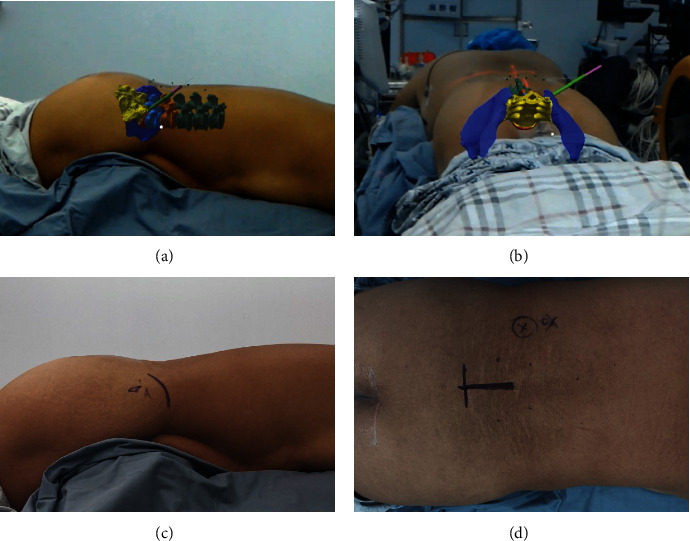
Integration of the three-dimensional (3D) model in the patient's body according to the external markers and internal anatomical landmarks. (a, b) The 3D model is matched with the patient's body from different directions. (c, d) Specific internal anatomical landmarks, such as the superior facet process of dorsal vertebra and the middle of the superior endplate, are identified by fluoroscopy and are used for matching with the 3D model.

**Figure 3:**
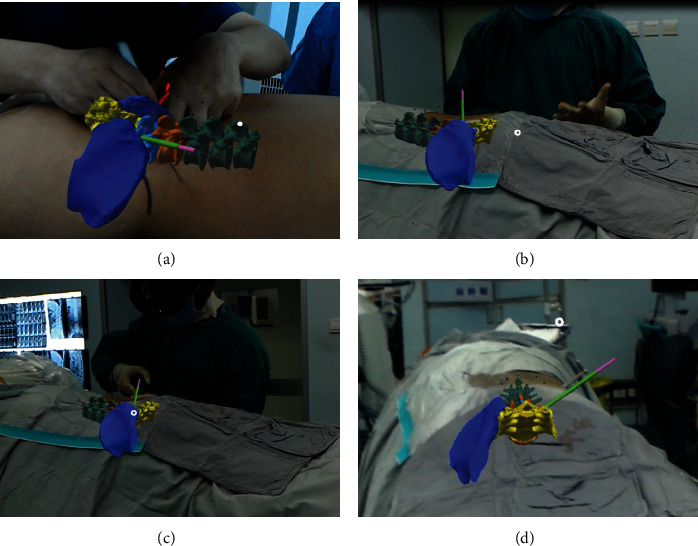
Four steps of the surgical procedure assisted with mixed reality technology: (a) marking; (b) needle insertion; (c) foraminoplasty; (d) positioning of the working sheath.

**Figure 4:**
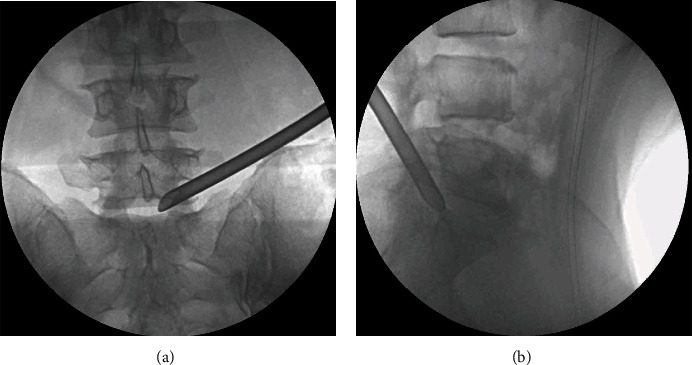
Final position of the working sheath confirmed with fluoroscopy: (a) anteroposterior radiograph; (b) lateral radiography.

The patient was positioned in the prone position on a radiolucent table. During the surgery, the 3D model was preliminarily integrated into the corresponding patient's body according to the external markers (Figures 2(a) and 2(b)). Standard anteroposterior and lateral views obtained with X-ray fluoroscopy were used to identify the superficial positions of the anatomical structures, and the precision of matching between the virtual model and the patient was assessed. The middle of the superior endplate and the superior facet process of the inferior vertebra were the most valuable anatomical landmarks (Figures 2(c) and 2(d)). If an unacceptable misalignment (≥5 mm) was present, the registration procedure was further optimized by incorporating the external and internal anatomical landmarks till an acceptable misalignment (<5 mm) was achieved [9]. Referring to the precisely matched virtual model in the MixR environment, the spinal level, midline, skin entry point, and puncture direction were marked on the skin. Needle insertion, foraminoplasty, and positioning of the working sheath were performed according to the preoperative plan under the guidance of MixR technology (Figure 3). The final positions of the spinal needle and working sheath were confirmed with fluoroscopy (Figure 4). Once the proper positioning of the working sheath was confirmed, endoscopic discectomy was performed after removing the OST-HMD.

### 2.5. Assessment of the Clinical Outcomes

For each patient, the demographic and disease-related data was collected from the medical records, including age, sex, body mass index (BMI), type of disc herniation, and surgical level; these data were also used to enroll patients into the matched conventional TPED group. The clinical outcomes were evaluated using the MacNab standard (excellent, good, fair, or poor) at the last follow-up examination [10]. A numerical rating scale (NRS) was used to assess the severity of leg or back pain preoperatively, on postoperative day 1, and at the last follow-up examination [11]. Furthermore, the Oswestry Disability Index (ODI) was used to evaluate the disability status [12]. The minimally important clinical difference (MICD) was defined as 3 for the NRS and 10% for the ODI. Exposure duration, from positioning to final fluoroscopy, and procedural duration, from the time of administration of local anesthesia to the end of the operation, were recoded and compared between the two groups.

### 2.6. Radiation Dose Measurement and Data Collection

The same surgeon performed all the procedures using a G-arm fluoroscope (Whale Healthcare, Beijing, China). The fluoroscopy time, tube voltage (TV), tube current (TC), and radiation dose for the anteroposterior and lateral views were directly read on G-arm fluoroscopy. The exposure duration was recorded in minutes.

### 2.7. Eye Fatigue Test

The operators completed the eye fatigue test before and after each operation. The eye fatigue test measured subjective fatigue from 0 to 7 points for tired eyes, sore or aching eyes, irritated eyes, dry eyes, eyestrain, hot or burning eyes, blurred vision, difficulty in focusing, and visual discomfort [13]. In addition, headache, dizziness, nausea, and decreased concentration were evaluated.

### 2.8. Statistical Analysis

Statistical analysis was performed using SPSS for Windows, version 20.0 (IBM Corp., Armonk, NY, USA). The continuous variables are expressed as the mean ± standard deviation (SD). For all patients, the NRS and ODI scores were compared preoperatively and postoperatively using paired *t*-tests. The two groups were compared using *t*-tests. *P* < 0.05 was considered statistically significant.

## 3. Results

### 3.1. General Information

There was no significant difference between the two groups with respect to the baseline parameters, including age, sex, BMI, and types and levels of disc herniation. The detailed information is listed in Table 1. Surgery was successfully completed for all patients, without dural rupture, nerve root injury, infection, poor healing of the incision, or serious allergy.

The clinical outcomes are listed in Table 2. Thirty-eight patients in the MixR-assisted TPED group and thirty-six cases in the conventional TPED group achieved excellent outcomes. Fair or poor outcomes were not observed at the last follow-up examination. There was no significant difference between the two groups with respect to the NRS score for leg or back pain, which significantly improved at 1 day postoperatively (*P* < 0.05) and at the last follow-up examination (*P* < 0.05), compared with the preoperative values. Significant improvement was observed in the ODI at the last follow-up visit compared with the preoperative value (*P* < 0.05). The NRS score and ODI did not differ significantly between the two groups at the last follow-up examination. All patients in both groups presented with greater improvement in the NRS score and ODI than in the MICD at the last follow-up visit compared with the preoperative values. The mean operation time in the MixR-assisted TPED and conventional TPED groups was 103 and 116 minutes, respectively (*P* = 0.01).

### 3.2. Fluoroscopic Data and Radiation Exposure

There was no significant difference in the mean TV and TC between the two groups. The durations of marking and needle insertion were shorter in the MixR-assisted TPED group than in the conventional TPED group (1.80 min *vs.* 2.75 min, *t* = 4.64, *P* < 0.001 and 5.39 min *vs.* 7.88 min, *t* = 3.58, *P* = 0.001, respectively). Similarly, with MixR assistance, the number of puncture attempts and radiation doses in the procedures of marking (3.93 *vs.* 6.88, *t* = 7.62, *P* < 0.001 and 3.01 mGy *vs.* 5.17 mGy, *t* = 7.38, *P* < 0.001, respectively) and needle insertion (3.41 *vs.* 6.33, *t* = 5.21, *P* < 0.001 and 2.73 mGy vs. 4.72 mGy, *t* = 4.56, *P* < 0.001) was significantly decreased. No significant differences were found with respect to the number of puncture attempts, duration, and radiation doses in the procedures of foraminoplasty and positioning of the working sheath between the two groups. With MixR assistance, the total radiation dose in the TPED procedure (13.59 mGy *vs.* 18.62 mGy, *t* = 3.96, *P* < 0.001) significantly decreased. The total number of puncture attempts and duration were obviously lower in the MixR-assisted TPED group than in the conventional TPED group (17.25 vs. 24.72, *t* = 5.01, *P* < 0.001 and 18.48 min *vs.* 23.87 min, *t* = 3.08, *P* = 0.003, respectively). The detailed information is displayed in Table 3.

### 3.3. Eye Fatigue Test

In the MixR-assisted TPED group, the eye fatigue scores were, in descending order, as follows: visual discomfort (2.7), headache (2.7), and blurred vision (2.6). In the conventional group, the main complaint regarding eye fatigue was decreased concentration (0.91). A few complaints were noted for other discomforts. We noted no significant differences in decreased concentration between the conventional TPED group and the MixR-assisted TPED group (*P* = 0.46).

## 4. Discussion

Recently, many guided techniques and strategies aimed at decreasing the radiation dose have been introduced [14]. MixR technology has been introduced in percutaneous kyphoplasty for the treatment of osteoporotic vertebral compression fractures. This suggests that MixR technology is practical and advantageous in percutaneous spinal operations. Thus, we introduced MixR technology in TPED with the aim of shortening the operating time and decreasing the radiation dose.

Using OST-HMDs, the operators can directly visualize the anatomical structure and virtual protruded disc. In our study, the operators made the needle coincide with that in the preoperative planned model; thus, the needle could reach the target quickly and accurately. Our results revealed that MixR assistance reduced the radiation dose and shortened the exposure duration during the procedures of marking and needle insertion. The number of puncture attempts and radiation dose were significantly reduced in the MixR-assisted TPED procedure compared to those in the conventional TPED procedure.

Foraminoplasty is strongly recommended in TPED for enlarging the intervertebral foramen near the facet joint, especially for complicated cases [15, 16]. During foraminoplasty, the target points need to be accurately reached through fluoroscopy. To reach the ideal target, fluoroscopy was routinely performed to identify the position of the reamer and the working sheath. Thus, the radiation dose and duration were not significantly reduced in the procedures of foraminoplasty and positioning of the working sheath in the MixR-assisted TPED group. Although the radiation dose is reduced and operating time is shortened with MixR technology [17], this technology still needs the assistance of conventional fluoroscopy. We also found greater displacement between superficial markers and anatomical structures in obese patients with a BMI greater than 30 kg/m^2^. It is necessary to ensure that the position of the patient during the CT scan is consistent with that assumed in the operation. Still, obese patients may experience more puncture attempts and radiation than others.

The incorporation of MixR technology in conventional fluoroscopy transformed the complicated surgical task into a simplified line alignment between the planned trajectory and working tools in multiple views, which helped surgeons reach the target site in a shorter time [18]. To easily and precisely match the 3D model with the anatomical spine, markers were extracted from the reconstructed 3D model. During the procedure of matching, the operators should endeavor to match multiple markers. After primary matching, standardized anteroposterior and lateral views were used to position the superficial projection of the anatomical structures, aiming the superficial projection at the 3D model from multiple perspectives aided in increasing the accuracy of the 3D model. We noted that the middle of the superior endplate and the superior facet process of the inferior vertebra were the ideal anatomical landmarks. Moreover, the operators should intermittently match the holographic model with the anatomical structures and superficial markers from multiple directions during the surgical procedure. We also found that the 3D holographic model reconstructed from flexion CT images provided greater accuracy and practicality in the TPED procedure than that obtained from conventional supine CT images. Flexion CT examination was considered the necessary component of the preoperative evaluation in patients who were diagnosed using magnetic resonance images. Thus, an additional CT scan was not required when MixR technology was used.

There are several potential advantages of MixR-assisted TPED. First, the 3D model and the procedural site are constantly present in the operator's field of view. The operators do not need to move their field of view away from the procedural site to obtain image guidance information. In addition, the change from the procedural site to the virtual model can be easily achieved by slightly raising the head. The transition from a classic clinical set-up to an OST-HMD display requires less adaptation than that required in other visualization models [19].

The introduction of MixR into TPED is aimed at not only decreasing the radiation dose and operation time but also reducing the operation difficulty and shortening the learning curve of residents [4]. In addition, we found great significance in MixR assistance in both the preoperative plan and the guidance of surgical procedures. Meanwhile, medical consumable material is not needed in MixR-assisted operations. Thus, the use of MixR technology cannot increase the expenses of patients and the government. Therefore, MixR technology has good cost-effectiveness in clinical practice and operations.

There are several limitations. The procedural time and radiation dose during working sheath placement may differ among patients with disc herniation at L5/S1 or L4/L5 [4]. Unfortunately, we cannot perform subgroup analysis according to the different levels because of the small sample size. Moreover, users may experience discomfort and eye fatigue when using the HoloLens for durations longer than half an hour. In addition, at the current early stage of development of this technology, only a few software programs of use are available to the surgeons. Both MixR system (Midivi) and StarAtlas 3.0 (Visual3D, Beijing, China) present the ability to manipulate and visualize holograms [20]. As the popularity of MixR technology increases, we expect that more surgically useful software will be developed [21].

## 5. Conclusions

This preliminary study shows the utility of MixR technology for image guidance in conventional TPED. The radiation exposure significantly decreased with MixR visualization guidance, and this technology serves as a valuable tool during the TPED procedure; however, the assistance of conventional fluoroscopy is still required.

## Figures and Tables

**Table 1:** Demographic and disease-related data of enrolled patients.

Group	MixR-assisted TPED group	Conventional TPED group	*P*
*N*	44	43	0.91
Male	24	24	
Female	20	19	
Age (years)	41.21 (17-64)	38.93 (19-62)	0.45
BMI (kg/m^2^)	24.27	24.19	0.86
Disc herniation type			
Central	16	14	0.98
Paracentral	20	20	
Foraminal	7	8	
Far-lateral	1	1	
Surgical level			
L3/4	4	1	0.37
L4/5	19	18	
L5/S1	21	24	

TPED: transforaminal percutaneous endoscopic discectomy; MixR: mixed reality; BMI: body mass index.

**Table 2:** Clinical outcomes of the two groups.

	MixR-assisted TPED group	Conventional TPED group	*P*
Preoperative NRS			
Back pain	3.57 ± 1.11	3.74 ± 1.07	0.45
Leg pain	6.57 ± 1.02	6.72 ± 0.91	0.46
Preoperative ODI (%)	63.02 ± 9.32	62.02 ± 8.22	0.60
Postoperative NRS			
Back pain	1.43 ± 0.90	1.60 ± 1.00	0.40
Leg pain	1.45 ± 0.98	1.70 ± 1.23	0.31
Postoperative ODI (%)	10.93 ± 5.02	9.56 ± 3.35	0.14
Improved NRS			
Back pain	2.14 ± 1.25	2.14 ± 1.44	0.99
Leg pain	5.11 ± 1.15	5.02 ± 1.52	0.75
Improved ODI (%)	52.09 ± 10.60	52.47 ± 9.52	0.86
Operation time (min)	103 ± 18	116 ± 29	0.01

TPED: transforaminal percutaneous endoscopic discectomy; MixR: mixed reality; NRS-LP: numerical rating scale for leg pain; NRS-BP: numerical rating scale for back pain; ODI: Oswestry Disability Index.

**Table 3:** Radiation exposure outcomes of the two groups.

	MixR-assisted TPED group	Conventional TPED group	*P*
Marking			
Puncture attempts	3.93 ± 1.88	6.88 ± 1.72	<0.001
Duration (min)	1.80 ± 0.67	2.75 ± 1.20	<0.001
Dose (mGy)	3.01 ± 1.36	5.17 ± 1.36	<0.001
Needle insertion			
Puncture attempts	3.41 ± 1.91	6.33 ± 3.18	<0.001
Duration (min)	5.39 ± 2.17	7.88 ± 4.10	0.001
Dose (mGy)	2.73 ± 1.71	4.72 ± 2.33	<0.001
Foraminoplasty			
Puncture attempts	6.09 ± 4.52	6.74 ± 6.81	0.60
Duration (min)	7.28 ± 5.42	7.95 ± 7.50	0.63
Dose (mGy)	4.80 ± 3.71	5.05 ± 5.07	0.80
Positioning sheath			
Puncture attempts	3.81 ± 1.42	4.77 ± 3.02	0.06
Duration (min)	4.01 ± 2.30	5.28 ± 3.73	0.06
Dose (mGy)	3.04 ± 1.38	3.68 ± 2.63	0.16
Total			
Puncture attempts	17.25 ± 4.28	24.72 ± 8.87	<0.001
Duration (min)	18.48 ± 6.38	23.87 ± 9.64	0.003
Dose (mGy)	13.59 ± 4.56	18.62 ± 7.07	<0.001

TPED: transforaminal percutaneous endoscopic discectomy; MixR: mixed reality.

## Data Availability

The data used to support the findings of this study are included within the article.
